# Prevalence and factors associated with the awareness of obstetric fistula among women of reproductive age in The Gambia: a multilevel fixed effects analysis

**DOI:** 10.1186/s12889-022-14107-7

**Published:** 2022-09-13

**Authors:** Agani Afaya, Alhassan Sibdow Abukari, Shamsudeen Mohammed

**Affiliations:** 1 Mo-Im Kim Nursing Research Institute, College of Nursing, Yonsei University, 50-1, Yonsei-ro, Seodaemun-gu, Seoul, 03722 South Korea; 2 Department of Nursing, School of Nursing and Midwifery, University of Health and Allied Sciences, Ho, Ghana; 3 Department of Nursing, School of Nursing & Midwifery, Wisconsin International University College, Postal Address, North Legon, Box LG 751, Accra, Ghana; 4 Department of Non-Communicable Disease Epidemiology, Faculty of Epidemiology and Population Health, London School of Hygiene & Tropical Medicine, London, UK

**Keywords:** Obstetric fistula, Reproductive age, Awareness, Gambia, DHS

## Abstract

**Background:**

An obstetric fistula is an inappropriate connection between the vagina, rectum, or bladder that results in faecal or urine incontinence. Young women from rural areas with poor socioeconomic situations and education are the majority of victims, which restricts their access to high-quality healthcare. Obstetric fistulas can have devastating effects on the physical health of affected women if they are not promptly treated. Inadequate awareness of the symptoms delays recognition of the problem, prompt reporting, and treatment. Women with poor awareness of the disorder are also more likely to develop complications, including mental health issues. Using data from a nationally representative survey, this study investigated the prevalence and factors associated with the awareness of obstetric fistula among women of reproductive age in The Gambia.

**Methods:**

This study used population-based cross-sectional data from the 2019–2020 Gambia Demographic and Health survey. A total of 11823 reproductive-aged women were sampled for this study. Stata software version 16.0 was used for all statistical analyses. Obstetric fistula awareness was the outcome variable. Multilevel logistic regression models were fitted, and the results were presented as adjusted odds ratios (aOR) with statistical significance set at *p* < 0.05.

**Results:**

The prevalence of obstetric fistula awareness was 12.81% (95%CI: 11.69, 14.12). Women aged 45–49 years (aOR = 2.17, 95%CI [1.54, 3.06]), married women (aOR = 1.39, 95%CI [1.04, 1.87]), those with higher education (aOR = 2.80, 95%CI [2.08, 3.79]), and women who worked as professionals or occupied managerial positions (aOR = 2.32, 95%CI [1.74, 3.10]) had higher odds of obstetric fistula awareness. Women who had ever terminated pregnancy (aOR = 1.224, 95%CI [1.06, 1.42]), those who listened to radio at least once a week (aOR = 1.20, 95%CI [1.02, 1.41]), ownership of a mobile phone (aOR = 1.20, 95%CI [1.01, 1.42]) and those who were within the richest wealth index (aOR = 1.39, 95%CI [1.03, 1.86]) had higher odds of obstetric fistula awareness.

**Conclusion:**

Our findings have revealed inadequate awareness of obstetric fistula among women of reproductive-age in The Gambia. Obstetric fistulas can be mitigated by implementing well-planned public awareness initiatives at the institutional and community levels. We, therefore, recommend reproductive health education on obstetric fistula beyond the hospital setting to raise reproductive-age women's awareness.

## Background

An obstetric fistula is an improper connection between the vagina, rectum, or bladder that causes urinary or faecal incontinence [1, 2]. In low- and middle-income countries (LMICs), obstetric fistulas typically result from prolonged and obstructed labour without timely access to high-quality medical intervention [3–5]. These types of fistulas were eradicated in high-income countries in the twentieth century, and fistulas in these places are usually caused by an injury during a surgical procedure, radiation therapy, or cancer [6]. In LMICs, obstetric fistulas due to childbirth remain as a major public health concern [1]. Most victims are young women from impoverished rural areas with low education and socioeconomic status, limiting their access to quality health care [5, 7]. For example, in sub-Saharan Africa (SSA) and South Asia, as many as two million young women live with the medical disorder, and an estimated 50,000 to 100,000 women are affected yearly [8]. In The Gambia, the prevalence ranges from 0.46 to 2.05 per 1000 women [9].

Obstetric fistulas can have devastating effects on the physical health of affected women if they are not promptly treated. For instance, obstetric fistulas can lead to chronic kidney diseases, recurrent infections of the urinary and reproductive tracts, secondary infertility, and painful genital sores [5]. In addition to the health consequences, obstetric fistulas impose social, psychological, and economic burdens on affected women and their families. Women with obstetric fistulas suffer discrimination, social stigma, shame, mental health problems, and generally poor quality of life. In many societies, women with obstetric fistulas are denied employment, abandoned by their husbands and families, and sometimes ostracised by their communities [3, 5, 10, 11]. As a result, the United Nations General Assembly has set a target for the eradication of obstetric fistula by the year 2030 [12].

Despite the negative effects of obstetric fistulas, it is often overlooked in policy discussions in developing countries, and research on the topic is scarce, probably because it affects marginalised women and girls disproportionately. In The Gambia, the United Nations Population Fund (UNFPA) is currently supporting many initiatives to improve the lives of women with obstetric fistulas, including the “Zero Fistula Gambia campaign”, which was launched in May 2022 [9]. In addition, the Ministry of Health of The Gambia and partners have implemented some interventions, including surgical repairs and access to skilled delivery for mothers with obstetric fistulas [13]. However, authorities are worried that there are no nationally representative data on the prevalence of obstetric fistula to support these initiatives and track progress. Similarly, a preliminary literature search showed that there are not many studies in The Gambia that used population-based surveys to assess the prevalence of obstetric fistula awareness and the characteristics of women at risk of not knowing the symptoms of obstetric fistula. Inadequate awareness of the symptoms delays recognition of the problem, prompt reporting, and treatment. In a recent analysis of cross-sectional data from fourteen sub-Saharan African countries, the researchers estimated the prevalence of obstetric fistula awareness in the region to be 37.9% [14]. Women with poor awareness of the disorder are also more likely to develop complications, including mental health issues.

Earlier studies in Ghana, Ethiopia, and Nigeria, have shown that living in an urban area, attending formal education, adequate attendance at antenatal care, delivery in a health facility, being employed, exposure to media, internet use, and high household income are positively associated with obstetric fistula awareness [15–18]. An estimate of the awareness of the disorder and the characteristics of women at risk of poor awareness is essential to planning national obstetric fistula educational campaigns and central to initiatives aimed towards eradicating fistulas. Increased awareness of obstetric fistulas may lead to increased financing from institutions for care and prevention and foster more collaborations in The Gambia at the community level. Therefore, this study aimed to investigate the prevalence and factors associated with the awareness of obstetric fistula among women of reproductive age in The Gambia using data from a nationally representative survey.

## Methods

### Study context

In The Gambia, the true burden of obstetric fistula is unknown due to a lack of nationally representative data. The prevalence of fistula, for instance, is based on proxy measurements such as treatment facilities, contextual information, and rates of maternal and perinatal mortality. The current national burden using data from these sources is between 335 to 1052 cases [9] as compared to the 2006 figure of 197 (0.5 per 1000) cases [19] of obstetric fistula. These estimates are not generally representative, and the actual burden might be higher than stated.

Nevertheless, The Gambia is considered among the 22 high-burdened countries in the world and was selected to train surgeons on obstetric fistula repair as part of the FIGO's Fistula Surgery Training program [20]. Efforts are also made by the government in collaboration with UNFPA to create awareness of the disease among reproductive-age women and enhance its repair to improve the quality of life of women. The Ministry of Gender, Children, and Social Welfare of Gambia has ensured the implementation of local programs and strategies aimed at tackling the complex circumstances and conditions that contribute to the development of obstetric fistulas in the country. In May 2022 the Zero Fistula Gambia campaign was launched, to raise public awareness of the condition and call for its eradication [9]. This campaign was targeted at achieving zero fistula cases in The Gambia by 2030 which collaborates with the international goals of UNFPA in ending obstetric fistula [9, 20]. The Gambia currently has three fistula centers, three fistula surgeons, and two FIGO-trained fellows. The facilities offering fistula repair are Edward Francis Small Hospital, Banjul, Bafrow Fistula center, Serekunda, and Kanifing General Hospital. Estimating the proportion of reproductive-aged women who are currently aware of the symptoms of obstetric fistula and the factors’ influencing awareness is necessary to assist these awareness programs and initiatives to track the progress and to improve public health education programmes.

### Source of data

The study used nationally representative data from the 2019–2020 Gambia Demographic and Health Survey (GDHS). The data collection for the GDHS was from November 21, 2019, to March 30, 2020. The Gambia Bureau of Statistics (GBoS) executed the survey in collaboration with the Ministry of Health (MoH) and with technical assistance from ICF through The DHS Program. Funding for the 2019–20 GDHS came from the United Nations Population Fund (UNFPA) and other agencies and organisations [2]. A multistage (two-stage) sampling design was employed to select households from the eight Local Government Areas (LGAs) in The Gambia for the survey. The first stage involved the stratification of the LGAs into rural and urban areas, based on an updated version of the 2013 Gambia Population and Housing Census (2013 GPHC), and the selection of 281 clusters (enumeration areas) with a probability proportional to their size within each sampling stratum. In the second stage, 25 households were selected from each cluster using a systematic sampling technique, resulting in a sample size of 7,025 households. Data were collected through face-to-face interviews with all women aged 15–49 who were permanent residents of the selected households or visitors who stayed overnight before the survey. Out of the 12,481 women aged 15–49 who were eligible for interviews in the selected households, 11,865 completed the interviews, yielding a response rate of 95% [2].

### Outcome variable

The primary outcome of this study was women’s awareness of obstetric fistula. Data on the outcome was extracted from the 2019–20 GDHS individual recode file which contained individual women’s data. The fistula module, which was included as part of the women’s questionnaire, asked women aged 15–49 years if they had ever heard of the phenomenon of urine or stool leaking from a woman’s vagina during the day and night, usually after a difficult childbirth, sexual assault, or pelvic surgery. In this analysis, the responses to the question (“have you ever heard about fistula?”) were dichotomous: *Yes* = ‘ever heard of fistula’ and *No* = ‘never heard of fistula’.

### Explanatory variables

The study considered 18 explanatory variables which were grouped into individual-level and household/community (contextual) level factors. The variables were determined based on the ecological model [21, 22] and through a review of previously published relevant studies, including systematic reviews and meta-analyses [17, 18, 23, 24]. Utilising an ecological model in a population-based study provides a unique contribution to knowledge on obstetric fistula awareness among reproductive-age women.

#### Individual level variables

Individual-level factors were the age of the woman, marital status, educational status, occupation, religion, health insurance coverage, parity, sexual experience, pregnancy status, ever terminated pregnancy, frequency of listening to radio, frequency of reading newspaper or magazine, frequency of watching television, owns a mobile telephone, and use of the internet.

The age of the women was categorised as 15–19, 20–24, 25–29, 30–34, 35–39, 40–44, and 45–49 while marital status was coded as never married, married, cohabitation, widowed, and divorced. Educational status was coded as no education, primary education, secondary education, and higher education; occupation was recoded as not working, managerial, clerical/sales, agricultural, services, and manual while religion was recoded as Christianity and Islam. Health insurance coverage was categorised as ‘no’ and ‘yes’, parity was recoded as null (0), 1–3, and ≥ 4 while the sexual experience was recoded as ‘not had sex’ and ‘had sex’. The pregnancy status of the women was categorised as no = ‘not currently pregnant’ and yes = ‘currently pregnant’ while pregnancy termination was coded as no = ‘never terminated pregnancy’ and yes = ‘ever terminated pregnancy’. Frequency of reading newspaper or magazine, frequency of watching television, and Frequency of listening to radio were categorised as ‘not at all’, ‘less than once a week’, and ‘at least once a week’. The use of the internet was categorized as ‘never’, ‘yes, last 12 months’, and ‘yes, before last 12 months.

#### Contextual level factors

The contextual level variables were selected based on the ecological model [21]. They included the sex of the household head, household wealth index, place of residence, and region. The sex of the household head was coded as ‘male’ and ‘female’ while the household wealth index was divided into five quantiles (poorest, poorer, middle, richer, and richest). The standard DHS data on ownership of household assets were used to compute the wealth index by selecting bicycles, television, house building materials, type of access to water, and sanitation facilities. The wealth index was generated from these assets through Principal Component Analysis (PCA). The PCA is a statistical procedure that is used to generate the wealth index by combining the household assets and grouped into five quantiles as stated above. The type of residence was coded as urban and rural while the region was categorized as Banjul, Kanifing, Brikama, Mansakonko, Kerewan, Kuntaur, Janjanbureh, and Basse [25].

### Statistical analysis

The analysis was conducted using Stata software version 16.0 (Stata Corporation, College Station, TX, USA). Descriptive statistics were used to present the distribution of obstetric fistula awareness across the categories of the explanatory variables, and chi-square test (χ2) was performed to determine the crude estimates of the association between obstetric fistula awareness and the explanatory variables. Because the 2019–20 GDHS nested women within households and households within clusters, we used a multilevel logistic regression to assess the association between the individual and contextual level factors and obstetric fistula awareness among the women for the multivariable analysis. A total of four models were built. The first model (Model O) was fitted as an empty model (random intercept) without predictors. We fitted the individual level variables into the second model (model I). The third model (model II) included the contextual level variables while in the final model (model III) we fitted all the explanatory variables against obstetric fistula awareness.

The multilevel logistic regression model comprised of fixed and random effects [26, 27]. Clusters were assumed as random effects to check for unexplained variability at the community level. The fixed effects showed the results of the association between the explanatory variables and obstetric fistula and were presented as adjusted odds ratios (aOR) with 95% confidence intervals. Intra Cluster correlation (ICC) was used to assess the random effects (measures of variation). The adequacy of the model was assessed using the loglikelihood ratio test while the Akaike's Information Criterion (AIC), and Bayesian Information Criteria (BIC) were used to evaluate model fitness. A multicollinearity diagnostic test was conducted and none of the explanatory variables had a high Variance Inflation Factor (VIF) necessary for exclusion (mini VIF = 1.02, max VIF = 3.47, mean VIF = 1.66). The sample was weighted (individual weight for women/1,000,000) to account for the unequal sampling of women from enumeration areas, and the survey set command in Stata was used in the analysis to account for the survey’s complex nature. Statistical significance was set at *p* < 0.05. We adhered to the guidelines outlined in the STROBE (Strengthening the Reporting of Observational Studies in Epidemiology) statement [28].

### Ethical approval

Ethical approval was not required for this secondary analysis. However, for the primary survey, the MEASURE DHS sought approval from the institutional review boards (IRBs) at ICF and The Gambia Government/Medical Research Council (MRC) Joint Ethics Committee in The Gambia before the commencement of data collection [2]. The MEASURE DHS approved our use of the 2019–20 GDHS data for this study.

## Results

A total of 11823 reproductive-age women were sampled for this study. Table 1 depicts the sociodemographic characteristics of the study sample and the proportion of women who had ever heard of obstetric fistula. At the individual level, 2,238 (18.9%) reproductive-age women were aged between 25–29 years, 7,480 (63.3%) were married, about 5,003 (42.3%) had secondary education, 4,753 (40.2%) were not working, while the majority 11,408 (96.5%) were Muslims. Approximately 11,499 (97.3%) were not covered by health insurance. About 879 (7%) of the women were pregnant at the time of data collection, 4,296 (36%) had never given birth while 9,843 (83%) had never terminated pregnancy. Most women (37.8%) listened to the radio at least once a week, 10,109 (85.5%) had never read a newspaper or magazine, 6,583 (55.7%) watched television at least once a week, 9,022 (76.0%) owned a mobile phone and about 7,291 (61.7%) used the internet in the 12 months before the survey. At the household/community level, most (78.0%) household heads were males and 23.9% of the women were in households in the richest wealth index. The majority (74.0%) of the women lived in urban areas (Table 1).

**Table 1:** Sociodemographic characteristics of respondents

Variables	Weighted N	Weighted %	Obstetric fistula awareness		
			**No (%)87.2%**	**Yes (%)12.8%**	**χ2** ***P*-value**
**Age (years)**					
15–19	2,630	22.2	93.7	6.3	< 0.001
20–24	2,172	18.4	89.2	10.8	
25–29	2,238	18.9	86.1	13.9	
30–34	1,606	13.6	83.5	16.5	
35–39	1,433	12.1	83.8	16.2	
40–44	1,028	8.7	83.7	16.3	
45–49	716	6.1	78.9	21.1	
**Marital status**					
Never married	3,686	31.2	91.7	8.3	< 0.001
Married	7,480	63.3	85.4	14.6	
Cohabitation	25	0.2	87.5	12.5	
Widowed	182	1.5	85.5	14.5	
Divorced	450	3.8	84.3	15.7	
**Educational status**					
No education	4,119	34.8	88.0	12.0	< 0.001
Primary	1,854	15.7	87.0	13.0	
Secondary	5,003	42.3	88.5	11.5	
Higher	847	7.2	69.5	30.5	
**Occupation**					
Not working	4,753	40.2	91.2	8.8	< 0.001
Managerial	579	4.9	66.9	33.1	
Clerical/sales	3,700	31.3	84.5	15.5	
Agricultural	1,962	16.6	87.0	13.0	
Services	320	2.7	90.0	10.0	
Manual	509	4.3	83.9	16.1	
**Religion**					
Christianity	415	3.5	87.2	12.8	< 0.001
Islam	11,408	96.5	87.1	12.9	
**Health insurance**					
No	11,499	97.3	87.2	12.7	< 0.001
Yes	324	2.7	78.4	21.6	
**Parity**					
Null	4,296	36.0	90.8	9.2	< 0.001
1–3	3,888	33.0	87.1	12.9	
4 and above	3,639	31.0	83.4	16.6	
**Sexual experience**					
Not had sex	3,386	29.0	91.8	8.2	< 0.001
Had sex	8,437	71.0	85.4	14.6	
**Pregnancy status**					
No or unsure	10,944	93.0	87.1	12.9	< 0.001
Yes	879	7.0	87.0	13.0	
**Ever terminated pregnancy**					
No	9,843	83.0	88.0	12.0	< 0.001
Yes	1,980	17.0	82.4	17.6	
**Frequency of listening to radio**					
Not at all	2,685	22.7	89.4	10.6	< 0.001
Less than once a week	4,674	39.5	87.1	12.9	
At least once a week	4,464	37.8	85.6	14.4	
**Frequency of reading newspaper or magazine**					
Not at all	10,109	85.5	87.9	12.2	< 0.001
Less than once a week	1,293	11.0	81.4	18.6	
At least once a week	421	3.5	77.9	22.1	
**Frequency of watching television**					
Not at all	2,110	17.8	87.2	12.8	0.046
Less than once a week	3,130	26.5	88.2	11.80	
At least once a week	6,583	55.7	86.4	13.6	
**Owns a mobile telephone**					
No	2,801	24.0	90.8	9.2	< 0.001
Yes	9,022	76.0	85.6	14.4	
**Use of internet**					
Never	4,083	34.5	89.0	11.0	< 0.001
Yes, last 12 months	7,291	61.7	85.6	14.4	
Yes, before last 12 months	449	3.8	87.0	13.0	
**Sex of household head**					
Male	9,198	78.0	86.9	13.1	0.212
Female	2,625	22.0	87.9	12.1	
**Wealth index**					
Poorest	1,998	17.0	87.4	12.6	< 0.001
poorer	2,132	18.0	89.1	10.9	
Middle	2,283	19.3	87.5	12.5	
Richer	2,579	21.8	87.9	12.1	
Richest	2,831	23.9	82.8	17.2	
**Type of place of residence**					
Urban	8,710	74.0	87.1	12.9	0.870
Rural	3,113	26.0	87.1	12.8	
**Region**					
Banjul	161	1.4	84.9	15.1	< 0.001
Kanifing	2,574	21.8	86.0	14.0	
Brikama	5,278	44.7	87.0	13.0	
Mansakonko	428	3.6	83.5	16.5	
Kerewan	1,127	9.5	89.6	10.4	
Kuntaur	522	4.4	83.4	16.6	
Janjanbureh	594	5.0	91.0	9.0	
Basse	1,136	9.6	89.0	10.9	

### Bivariate association between obstetric fistula awareness and explanatory variables

The overall prevalence of obstetric fistula awareness among reproductive-age women in The Gambia was 12.8% **(**95% CI: 11.7 – 14.0**).** The age (years), marital status, educational level, occupation, religion, health insurance coverage, parity, sexual experience, currently pregnant, ever terminated pregnancy, frequency of listening to radio, frequency of watching television, owns a mobile telephone, Use of internet, Wealth index, type of place of residence, and region were statistically associated with the awareness of obstetric fistula among reproductive-age women in the bivariate analysis (Table 1).

### Random effects (measures of variations) results

From Table 2, the empty model showed a substantial variation in the likelihood of obstetric fistula awareness among women in The Gambia across the primary sampling units **(**PSUs) clustering (σ2 = 0.53 95% CI [0.40–0,70]). The empty model (Model 0) indicated that 13.9% of the variation in obstetric fistula awareness among women in The Gambia was attributed to the variation between-cluster characteristics, i.e., (ICC = 0.1395172). The variation between clusters decreased slightly to 13.7% in Model I, representing only the individual level model (Model I). In the contextual level only model (Model II), the ICC decreased to 12.5%. There was further slight decline (12.4%) in the ICC in the complete Model (model III). This further emphasize that the variations in the likelihood of obstetric fistula awareness among women in the Gambia are attributed to the clustering differences within PSUs. The AIC value showed a successive reduction, which means a substantial improvement in each of the models over the previous model and also affirmed the goodness of Model III developed in the analysis. Also, the best fit model was determined by the highest loglikelihood (-4174.9847) value among the models. Therefore, the complete model (Model III) consisting of all the explanatory variables was selected to predict obstetric fistula awareness among reproductive-aged women in the Gambia.

**Table 2:** Fixed and random effects multilevel analysis of factors associated with obstetric fistula awareness among women of reproductive age

Variable	Model O	Model I aOR [95% CI]	Model II aOR [95% CI]	Model III aOR [95% CI]
**Fixed effect results**				
**Age (years)**				
15–19		–-		–-
20–24		1.26[0.99, 1.59]		1.25 [0.99, 1.59]
25–29		1.47** [1.16, 1.94]		1.48**[1.14, 1.92]
30–34		1.73***[1.29, 2.31]		1.69*** [1.26, 2.25]
35–39		1.7***[1.25, 2.31]		1.65**[1.21, 2.25]
40–44		1.73**[1.24, 2.40]		1.69** [1.22, 2.36]
45–49		2.27***[1.61, 3.19]		2.17***[1.54, 3.06]
**Marital status**				
Never married		–-		–-
Married		1.398* [1.04, 1.88]		1.39*[1.04, 1.87]
Cohabitation		1.030[0.21, 5.10]		1.00 [0.20, 4.98]
Widowed		1.232 [0.71, 2.13]		1.24 [0.72, 2.14]
Divorced		1.258 [0.84, 1.89]		1.24 [0.83, 1.86]
**Educational status**				
No education		–-		–-
Primary		1.26** [1.06, 1.50]		1.25* [1.05, 1.49]
Secondary		1.29**[1.08, 1.52]		1.27** [1.07, 1.51]
Higher		2.94***[2.18, 3.95]		2.80***[2.08, 3.79]
**Occupation**				
Not working		–-		–-
Professionals/Managerial		2.34***[1.75, 3.12]		2.32***[1.74, 3.10]
Clerical/sales		1.33*** [1.13, 1.56]		1.34***[1.14, 1.58]
Agricultural		1.18[0.99, 1.42]		1.19 [1.00, 1.44]
Services		1.04[0.68, 1.58]		1.07[0.70, 1.63
Skilled/unskilled workers		1.65**[1.21, 2.26]		1.74***[1.26, 2.38]
**Religion**				
Christianity		–-		–-
Islam		0.81[0.57, 1.15]		0.79 [0.56, 1.14]
**Health insurance**				
No		–-		–-
Yes		1.02[0.72, 1.46]		0.99[0.70, 1.42]
**Parity**				
0		–-		–-
1–3		0.95[0.75, 1.21]		0.96[0.76, 1.23]
4 and above		1.19[0.91, 1.57]		1.22 [0.93, 1.62]
**Sexual experience**				
Not had sex		–-		–-
Had sex		1.02 [0.72, 1.44]		1.02 [0.72, 1.44]
**Ever terminated pregnancy**				
No		–-		–-
Yes		1.23**[1.06, 1.42]		1.22** [1.06, 1.42]
**Frequency of listening to radio**				
Not at all		–-		–-
Less than once a week		1.140 [0.97, 1.34]		1.16 [0.99, 1.37]
At least once a week		1.18*[1.01, 1.39]		1.20*[1.02, 1.41]
**Frequency of reading newspaper or magazine**				
Not at all		–-		–-
Less than once a week		1.17 [0.95, 1.45]		1.16[0.94, 1.44]
At least once a week		0.99 [0.70, 1.40]		0.96[0.68, 1.36]
**Frequency of watching television**				
Not at all		–-		–-
Less than once a week		0.89 [0.75, 1.07]		0.91[0.76, 1.10]
At least once a week		1.00[0.84, 1.19]		0.98[0.81, 1.19]
**Owns a mobile telephone**				
No		–-		–-
Yes		1.19*[1.01, 1.41]		1.20*[1.01, 1.42]
**Use of internet**				
Never		–-		–-
Yes, last 12 months		1.04 [0.89, 1.22]		1.03 [0.88, 1.22]
Yes, before last 12 months		1.09 [0.80,1.48]		1.105[0.81,1.51]
**Wealth index**				
Poorest			–-	–-
Poorer			0.93 [0.77, 1.13]	0.92[0.75, 1.12]
Middle			1.18[0.95, 1.47]	1.11 [0.88, 1.41]
Richer			1.23[0.96, 1.58]	1.10 [0.84, 1.46]
Richest			1.80***[1.39, 2.33]	1.39* [1.03, 1.86]
**Region**				
Banjul			–-	–-
Kanifing			0.90 [0.62, 1.33]	0.91 [0.62, 1.35]
Brikama			0.93[0.64, 1.35]	0.92[0.63, 1.35]
Mansakonko			1.46[0.93, 2.30]	1.48[0.93, 2.36]
Kerewan			0.76[0.49, 1.18]	0.78 [0.50, 1.24]
Kuntaur			1.60* [1.01, 2.53]	1.83*[1.15, 2.93]
Janjanbureh			0.73[0.45, 1.16]	0.76 [0.48, 1.24]
Basse			0.90[0.59, 1.39]	1.05 [0.68, 1.63]
**Random effect model**				
PSU variance (95% CI)	0.53 [0.40–0,70]	0.52 [0.39–0.69]	0.47 [0.35–0.62]	0.46 [0.35–0.62]
ICC	0.1395172	0.1375541	0.125597	0.1249966
Wald chi-square	Reference	410.60***	56.15**	439.60***
**Model fitness**				
Log-likelihood	-4410.7843	-4191.4134	-4382.9262	-4174.9847
AIC	8825.569	8452.827	8791.852	8441.969
BIC	8840.324	8711.05	8887.764	8781.348

Exponentiated coefficients; 95% confidence intervals in brackets; *aOR* adjusted odds ratios, *CI* Confidence Interval; ^*^ *p* < 0.05, ^**^ *p* < 0.01, ^***^ *p* < 0.001; –- = Reference category; *ICC* Intra-Class Correlation; *AIC* Akaike’s Information Criterion, *BIC* Bayesian information criterion

### Determinants of obstetric fistula awareness among reproductive-age women in the Gambia

#### Fixed effects (measures of associations) results

Table 2 depicts results from the multilevel analysis on the determinants of obstetric fistula awareness among women in The Gambia after adjusting for other factors. In the final model, we found that increasing age was associated with higher awareness of obstetric fistula. In particular, women between the ages of 45 and 49 years were two times more likely to have higher awareness of obstetric fistula than those aged 15–19 years (aOR = 2.17, 95% CI [1.54,3.06]). Women who were married (aOR = 1. 39, 95**%** CI [1.04,1.87]) had higher odds of being aware of obstetric fistula than those who were not married. Women with higher education (aOR = 2.81, 95% CI [2.08,3.79]) were more likely to be aware of obstetric fistula than those without education. Also, women who worked as professionals/occupied managerial positions (aOR = 2.32, 95% CI [1.74,3.10]) had higher odds of obstetric fistula awareness than those with any occupation. Reproductive age women who had ever terminated pregnancy (aOR = 1.22, 95% CI [1.06,1.42]) were more likely to be aware of obstetric fistula than their counterparts. Women who listened to the radio at least once a week (aOR = 1.20, 95% CI [1.02,1.41]) had a higher odds of obstetric fistula awareness compared to those who have never listened to the radio. Reproductive age women who owned a mobile phone (aOR = 1.20, 95% CI [1.01,1.42]) and those who were within the richest wealth index (aOR = 1.39, 95% CI [1.03,1.86]) had higher odds of obstetric fistula awareness.

## Discussion

### Summary of findings

We investigated the prevalence and women's awareness of obstetric fistula in The Gambia using data from a nationally representative survey. Our analysis revealed that only 12.8% of the reproductive-age women included in this study were aware of obstetric fistula. Older women, women who ever terminated a pregnancy, and married women were more likely to be aware of obstetric fistula than their counterparts. The results of this study show that women who attained primary, secondary, or higher education had higher awareness of obstetric fistula than those who never attended school. Furthermore, women were more likely to be informed of obstetric fistula if they lived in a high-income household, worked as professionals/occupied managerial positions, listened to the radio at least once a week, and owned a mobile phone.

### Comparison with other studies

The awareness level of obstetric fistula in this study is lower than the prevalence reported in several previous studies in SSA [15–18, 29], highlighting the need for obstetric fistula education among reproductive age women in The Gambia. However, in line with our findings, a study in Ethiopia reported high awareness of obstetric fistula among older women and women with higher education [17]. In the Ethiopian study, media exposure and household income were also associated with higher odds of obstetric fistula awareness [17]. Older women were better informed about obstetric fistula in the present study probably because they are much more likely to have experienced multiple births that exposed them to education on birth complications and reproductive health issues, including education on obstetric fistulas at antenatal and postnatal clinics. For example, in Nigeria, women with previous childbirth experience had about two times higher awareness of obstetric fistula than those with no birth experience [18]. It is also possible that older women had higher awareness of obstetric fistula in this study because, in most African countries, including The Gambia, older women play a critical role as birth attendants and the first point of call when younger women experience complications after birth, especially if the delivery was conducted outside a healthcare facility [30]. In addition, older women are more likely to have attained higher education than younger women. Education increases women's access to and utilisation of healthcare information [31–33]. As a result, better-educated women tend to be more informed about their reproductive health than uneducated women. This further explains why mothers with any level of education had a higher awareness of fistula than women with no education in this study. Balcha et al., reported similar findings in Ethiopia [15].

Furthermore, a study in Nigeria found that women who had ever terminated pregnancy were more likely to be aware of obstetric fistula, which is consistent with our finding [18]. Education and counselling of women seeking an abortion on the possible complications of the procedure, particularly if instruments are used, may explain the higher awareness of obstetric fistula among women who ever terminated a pregnancy. Listening to the radio and ownership of a phone were also associated with higher awareness of obstetric fistula in the Nigerian study [18]. Exposure to reproductive health information may explain the high awareness of obstetric fistula among women who listen to the radio at least once a week since education on pregnancy and pregnancy-related complications is discussed on the radio.

In congruence with the results of this study, Azanu et al., found that, in Ghana, women engaged in skilled and semiskilled occupations had higher odds of obstetric fistula awareness [16]. However, contrary to our findings, the Ghanaian study found no evidence of an association between marital status and awareness of obstetric fistula; probably because, unlike the present study, the Ghanaian study was facility-based, included a relatively small sample (*n* = 393), and was restricted to only women attending prenatal clinic [16]. Studies have shown that married women are more likely to seek maternal healthcare services such as antenatal and postnatal care than unmarried women [34–36]. The better healthcare-seeking behaviour of married women is probably the reason for the high awareness of obstetric fistula since education provided during these services includes birth complications.

### Implications for policies and research

It is worrying that only a small proportion of reproductive-aged women were aware of obstetric fistula in The Gambia. The capacity to recognise the complication is critical to early diagnosis and treatment. Postpartum women may live with obstetric fistula for a long time without reporting it to healthcare providers because of the inability to recognise it [37]. The findings of this study could be used to improve existing reproductive health education programs on obstetric fistula in The Gambia. The obstetric fistula education programs could be intensified in healthcare facilities, at community centres, women groups, on radio, and television. It has been shown that educational campaigns on obstetric fistula can increase awareness of the problem [38, 39]. Policies to incorporate and prioritise fistula education could also consider the findings from this study to increase the awareness of obstetric fistula in The Gambia [40]. The education should include information on the causes, early signs of a fistula, places to report for interventions, and what treatments are available. Culturally appropriate posters and booklets with simple clear messages and images would be effective to carry across the message. Women with adequate awareness should be included in the education as awareness does not necessarily translate into appropriate obstetric fistula knowledge. For instance, in a Malawian study, women with high awareness of obstetric fistula associated fistulas with sexually transmitted diseases, women's laziness during labour, witchcraft, and the husband's infidelity [41]. To ensure the success of obstetric fistula education campaigns, traditional birth attendants must be included and actively involved in the campaigns since they are critical providers of reproductive health services, particularly in rural communities [30, 38, 42]. If given adequate training, TBAs can serve as facilitators of the fistula education campaigns working with professional healthcare providers to educate reproductive aged women. TBAs are also actively engaged in religious, gender, and cultural wellbeing of their society [30] and could assist to correct misperceptions about obstetric fistula and delays in seeking care that is fostered by cultural and religious practices. Encouragement and support of women to attain high education could increase their awareness of obstetric fistula as higher education is associated with higher access and utilisation of health information and services [31–33]. Future studies should assess women's knowledge of the early signs and risk factors for obstetric fistulas, as awareness does not necessarily mean sufficient knowledge.

## Strengths and limitations

The main strength of this study is the use of a large nationally representative sample that makes our results representative of reproductive-age women in The Gambia. In addition, the multilevel analysis approach we adopted ensured that the hierarchical nature of the survey was accounted for in the analysis, and the results are less likely to be influenced by the clustering of participants in enumeration areas. Recall bias is the study's major limitation as data on covariates were collected from women retrospectively. Also, the data source lacked variables on cultural practices and other factors that can influence women's awareness of obstetric fistula.

## Conclusion

Our findings have revealed inadequate awareness of obstetric fistula among reproductive-age women in The Gambia. The few women with adequate awareness were older, had attained some formal education, lived in households with high income, owned a mobile phone, listened to the radio, and were married. Women's access to and use of healthcare information is improved by regular education. It is also likely that rising awareness of obstetric fistula is attributable to women seeking abortions being counseled about the risks of the procedure. Implementing well-planned public awareness programs at the institutional and community levels may help to prevent obstetric fistulas. We, therefore, recommend reproductive health education on obstetric fistula beyond the hospital setting to raise reproductive-age women's awareness.

## Data Availability

The datasets used for this study is openly available and can be accessed via https://dhsprogram.com/data/.

## Abbreviations

GBoS: The Gambia Bureau of Statistics
GDHS: Gambia Demographic and Health Survey
aOR: Adjusted odds ratio
CI: Confidence Interval
ICC: Intra-Class Correlation
AIC: Akaike’s Information Criterion
BIC: Bayesian information criterion
MRC: Medical Research Council
LGAs: Local Government Areas
VIF: Variance Inflation Factor
PCA: Principal Component Analysis
MOH: Ministry of Health
UNFPA: United Nations Population Fund
GPHC: Gambia Population and Housing Census
SSA: Sub-Saharan Africa
LMICs: Low and Middle Income Countries
